# Addressing Operational Challenges Faced by COVID-19 Public Health Rapid Response Teams in Non–United States Settings

**DOI:** 10.1017/dmp.2020.487

**Published:** 2020-12-22

**Authors:** Puneet Anantharam, Adela Hoffman, Michelle Noonan, Dante Bugli, Laura Pechta, Jennifer Bornemann, Kerton R. Victory, Ashley L. Greiner

**Affiliations:** 1 Centers for Disease Control and Prevention, Division of Global Health Protection, Emergency Response and Recovery Branch, Atlanta, Georgia, USA; 2 Centers for Disease Control and Prevention, Office of the Chief Operating Officer, Office of Safety, Security & Asset Management, Atlanta, Georgia, USA; 3 Centers for Disease Control and Prevention, Division of Global Health Protection, Office of the Director, Atlanta, Georgia, USA

**Keywords:** rapid response teams, COVID-19, emergency responders, emergency preparedness, disease outbreaks

## Abstract

The coronavirus disease 2019 (COVID-19) global response underscores the need for a multidisciplinary approach that integrates and coordinates various public health systems—surveillance, laboratory, and health-care systems/networks, among others—as part of a larger emergency response system. Multidisciplinary public health rapid response teams (RRTs) are one mechanism used within a larger COVID-19 outbreak response strategy. As COVID-19 RRTs are deployed, countries are facing operational challenges in optimizing their RRT’s impact, while ensuring the safety of their RRT responders. From March to May 2020, United States Centers for Disease Control and Prevention received requests from 12 countries for technical assistance related to COVID-19 RRTs and emergency operations support. Challenges included: (1) an insufficient number of RRT responders available for COVID-19 deployments; (2) limited capacity to monitor RRT responders’ health, safety, and resiliency; (3) difficulty converting critical in-person RRT operational processes to remote information technology platforms; and (4) stigmatization of RRT responders hindering COVID-19 interventions. Although geographically and socioeconomically diverse, these 12 countries experienced similar RRT operational challenges, indicating potential applicability to other countries. As the response has highlighted the critical need for immediate and effective implementation measures, addressing these challenges is essential to ensuring an impactful and sustainable COVID-19 response strategy globally.

The ongoing outbreak of coronavirus disease 2019 (COVID-19) has spread globally, resulting in the World Health Organization’s (WHO) declaration of a pandemic in March 2020.^1,2^ The COVID-19 response to date has underscored the need for a multidisciplinary public health approach that integrates and coordinates surveillance, laboratory, and health-care systems/networks, among others, as part of a larger emergency response system. Public health rapid response teams (RRTs) are one mechanism within a larger emergency response strategy that can be used in a COVID-19 outbreak to ensure an efficient and effective response.^3-7^ The United States Centers for Disease Control and Prevention (CDC) defines a public health RRT as a multidisciplinary team that is trained and equipped with the capacity to rapidly deploy to a public health emergency in coordination with other response efforts.^3^ COVID-19 RRTs are often composed of case management, epidemiology, laboratory, risk communication, social mobilization, and infection prevention and control specialists.^6^ As COVID-19 RRTs are deployed, countries face operational challenges in optimizing their RRT’s impact while ensuring the safety of their RRT responders.

From March to May 2020, the CDC received requests from 12 countries for technical assistance related to COVID-19 RRTs and emergency operations support. The requests originated from foreign governments directly or through their respective CDC Country Offices. To ensure disclosure of challenges faced, countries’ anonymity has been maintained. The countries are geographically and economically diverse, with a 2017 gross domestic product per capita range of $1,000-$50,000 USD.^8^ Before the COVID-19 pandemic, most countries had been developing and formally instituting their emergency response capacity under global initiatives such as the Global Health Security Agenda (GHSA).^9-12^ Of the 12 countries, 6 had received 2015 GHSA funding and technical support as designated Phase 1 countries, and 3 received technical assistance as Phase 2 countries.^12^ All countries reported having national RRTs established before COVID-19 but with variable functionality; only 50% had standard operating procedures (SOPs) encompassing all RRT management processes, including administration, budget, staffing, roster maintenance, training, pre-deployment, deployment, and post-deployment processes.^3^

Regardless of variations in RRT infrastructure, countries faced common RRT operational challenges during the COVID-19 response. Major challenges identified include: (1) an insufficient number of RRT responders available for COVID-19 deployments; (2) limited capacity to monitor RRT responders’ health, safety, and resiliency; (3) difficulty converting critical in-person RRT operational processes to remote information technology (IT) platforms; and (4) stigmatization of RRT responders hindering COVID-19 interventions. Though not all unique to the COVID-19 response, these RRT operational challenges have impacted countries’ COVID-19 response activities.^13-17^ The following sections describe these operational challenges as well as selected recommendations to address them (Table 1). With minimal guidance currently available to address these challenges, the recommendations incorporate existing management science principles of capacity design with more than 20 CDC and WHO guidance documents, as well as CDC subject-matter expert input from multiple disciplines covering RRT management, social mobilization, risk communication, responder resiliency, and occupational safety.^6,7,18-30^

**Table 1.** Challenges and recommendations for coronavirus disease 2019 (COVID-19) public health rapid response teams (RRTs)

	Challenge	Recommendation
COVID-19 RRT responder availability	Responders with higher risk for severe COVID-19 illness	Recruit and virtually onboard new responders Have existing RRT responders take on multiple roles Assign higher risk responders to emergency coordination unit roles
	Concerns for contracting the virus and/or being a carrier	Strengthen administrative and safety RRT standard operating procedures (SOPs) Explain COVID-19 safety measures during RRT recruitment Provide COVID-19 resources to RRT responder’s families
COVID-19 RRT responder health, safety, and resiliency	Deficient or nonexistent RRT safety monitoring protocols	Expand pre-deployment processes to include COVID-19 safety considerations Assess proper personal protective equipment (PPE) use Implement COVID-19 health monitoring processes Deploy field safety experts
	Disruption of routine responder stress mechanisms/networks	Institute a resiliency program Facilitate communication and connection of returning responders Have mental health professionals participate in post-deployment debriefs
COVID-19 remote RRT operations	Difficulty converting in-person RRT operational processes to remote platforms	Procure necessary information technology (IT) infrastructure Adapt critical operation SOPs to remote COVID-19 environment Update pre-deployment and just-in-time (JIT) training processes Use remote platforms during the post-deployment period
COVID-19 RRT stigma	Stigma targeting COVID-19 RRT responders	Include information, tools, and trainings about COVID-19 stigma Engage with local stakeholders who can publicly support RRT responders and liaise with the community Conduct sensitization campaigns and provide communication materials to household members regarding RRT responders returning home

## COVID-19 RRT Responder Availability

During the COVID-19 response, a major RRT operational challenge has been an insufficient number of RRT responders available for deployments. This insufficiency was relative to the countries’ specific situation and response context, including the COVID-19 transmission scenario and human capacity availability. Factors underlying this challenge include: (1) the exclusion of individuals with a higher risk for severe COVID-19 illness (eg, individuals ≥65 years of age, individuals with certain underlying health conditions) from deployment for their health and safety, and (2) RRT responder concerns regarding the elevated risk of contracting the virus and/or being carriers when returning home to their households.^31-33^

To overcome a limited COVID-19 responder pool, recruitment of new responders along with the use of a COVID-19 just-in-time (JIT) training, in lieu of a standard RRT orientation training, may be considered.^6^ JIT trainings are shorter, subject-matter focused, and response specific, allowing for quicker onboarding of RRT responders.^3,6^ Identifying and onboarding new responders during an active response can be time and resource intensive; using existing RRT responders who can take on multiple COVID-19 RRT roles in the field provides an alternative option to address RRT workforce constraints. Roles that have substantial cross-over with other RRT responder roles due to inherently similar skillsets (eg, risk communication and social mobilization roles; case management and infection prevention and control roles) can provide greater deployment coverage in the field with fewer personnel.^6^ Deploying individuals in dual roles can be facilitated by means of pre-deployment, role-specific JIT trainings focused on their translatable expertise.

RRT responders considered to be at higher risk for severe illness can still be used in the response by assigning those individuals to the country’s emergency response coordination unit instead of the field.^26,31^ As a trained response workforce, they can take on response coordination roles and/or provide remote technical assistance to their RRT counterparts in the field.^3^ If a country has the IT infrastructure for teleworking, another option may be to have the RRT responder at higher risk for severe illness support the emergency coordination unit remotely.

RRT SOPs addressing health, medical evacuation, and insurance can be strengthened to address RRT responder concerns regarding COVID-19 infection.^3^ COVID-19 RRT safety measures (described in the next section) should be promoted and explained during RRT recruitment. Extension of these resources to RRT responders’ families in the event they become infected can be considered to address concerns about ongoing COVID-19 transmission.^3,6^ If financially and logistically feasible, temporary post-deployment lodging for RRT responders can be provided to protect household members who may be at higher risk for severe illness.

## COVID-19 RRT Responder Health, Safety, and Resiliency

The second major RRT operational challenge during the COVID-19 response is the limited capacity to monitor RRT responders’ health, safety, and resiliency. Underlying this challenge has been: (1) deficient or nonexistent RRT safety monitoring protocols meeting the rigorous safety measures requisite when deploying for COVID-19, and (2) the disruption of responder stress management due to intense COVID-19 safety measures limiting social interaction and physical support, which underlie typical coping mechanisms.^13,34,35^

To address this challenge, pre-deployment processes can be expanded to include COVID-19-specific safety considerations. An example might be instituting and/or expanding pre-deployment medical examinations to include screening for severe COVID-19 illness risk factors, ensuring those at higher risk are not deployed.^3,6,31^ Additionally, assessments should be implemented for proper donning and doffing of personal protective equipment (PPE) and ensuring the proper fit and use of respirators, if required.^23,24^ While assessments can be done remotely using videos and/or interactive sessions, PPE donning and doffing and respirator use can be intricate processes; practice and confirmation of proper use should occur before deploying and monitored during deployment, if feasible.

To ensure RRT responder safety during deployment and post-deployment, COVID-19–specific health monitoring processes can be implemented to immediately identify when an RRT responder feels ill, reduce delays in medical care, and limit onward transmission. Ideally, the SOP for this monitoring process would delineate the personnel responsible for monitoring, required IT systems, reporting frequency, RRT equipment (eg, thermometers) provision, and the downstream processes that occur when COVID-19 signs or symptoms develop (eg, isolation, testing, evacuation, and treatment procedures). Deployment of field safety experts trained in safety, security, resilience, and/or health monitoring can also be considered, if such experts are available. In addition to safety risk assessments, these deployed field safety experts can provide crucial monitoring and directly address issues of distress, burnout, stigma, and fatigue that may affect an RRT responder’s health while in the field.^34^

To promote and support RRT responders’ coping mechanisms during COVID-19, resources may be allocated to institute a resiliency program that provides emotional support, facilitates connectedness, and encourages recognition of resiliency issues. Particularly with COVID-19 social distancing measures instituted, facilitating the communication and connection of recently returned responders (eg, by means of a “buddy” system) can be considered. Regardless of the RRT responder support provided, mental health professionals can also participate in post-deployment debriefs to identify any potential stressors, provide proactive emotional support, and share behavioral health resources.^36^

## COVID-19 Remote RRT Operations

The third major challenge identified is the difficulty converting critical in-person RRT operational processes to remote IT platforms during the COVID-19 response. Underlying this challenge is the unprecedented reliance on IT infrastructure and remote processes as a result of the rigorous COVID-19 social distancing requirements.^37-39^ RRT pre-deployment briefings, JIT trainings, and post-deployment debriefs—essential processes to ensure a prepared, effective, and supported workforce—are traditionally conducted in-person and, thus, have been specifically impacted by this challenge.

In addition to procuring the necessary IT infrastructure to remedy this issue, the SOPs for these critical RRT operations should be adapted to the remote COVID-19 environment. SOPs must identify who will facilitate these operations, what modality will be used (eg, group teleconference vs individual phone calls), how resources/files will be shared, and what COVID-19 information is pertinent to include. For alternative training modalities, keys to success weigh heavily on their integration with traditional education and existing training programs.^40^ Many RRT management programs already use technology platforms to communicate with and train RRT responders. If adequate IT infrastructure cannot be instituted, shifting from remote group interaction to individual phone calls may be an option; however, plans should account for this method being more time and resource intensive.

Acknowledging the rapidly evolving COVID-19 guidance, processes to regularly update the pre-deployment briefing and JIT training content should be considered.^6^ For example, an electronic resource library can be a tool to compile and provide updated resources for RRT responders. Standard materials should be used to ensure RRT responders receive uniform and applicable information with the potential for downstream implications for RRT readiness and impact.^3,6^

For the post-deployment period, debrief SOPs will need to consider a remote platform to ensure there is no delay in collecting responder feedback due to any instituted post-deployment self-isolation periods. Responder feedback should be elicited immediately upon return from the field, as this critical information is used to inform ongoing response efforts.

## COVID-19 RRT Stigma

Stigma targeting COVID-19 RRTs is the final RRT operational challenge, hampering the implementation of COVID-19 response activities. Although prevalent in other outbreaks, the novelty of the virus and rapidly changing information during the COVID-19 response has made stigma a pervasive issue notably in the community and within RRT responder households.^13,41,42^ Factors contributing to this stigma include fear, lack of knowledge, and the perception of RRT responders as COVID-19 carriers.^33,43-46^ Stigma can affect acceptance of COVID-19 interventions, and may contribute to RRT responder stress, fatigue, and burnout.^33,34^ Additionally, responders may experience avoidance, a form of stigma, within their own household and community.^32,35^

To address community stigma, the pre-deployment briefing should include information about COVID-19 stigma as well as tools and/or JIT training to better equip responders in addressing it. This can include the provision of communication materials for responders to distribute, guidance on how to engage with the community, answers to commonly asked COVID-19 questions, and talking points to communicate the RRT’s mission.^21,22^ If possible, responders can engage with locally trusted community leaders, health-care workers, and/or local organizations who can publicly support the RRT responders and liaise with the community.^47,48^ Due to the unprecedented nature of the COVID-19 pandemic, there has been an influx of information in many communities where RRTs deploy.^49^ Hence, there is a need for RRT members to engage with partners and “ground truth” to discover potential reasons behind the stigma of RRTs. This also underscores the critical need for RRT responders to receive JIT training on COVID-19–specific risk communication and community engagement.^6^ Social mobilization efforts should be tailored to the COVID-19 transmission scenario, the specific location, and needs of the community being supported.

Overcoming stigma in responder households is critical in transitioning a responder back to their normal routine and to their personal resiliency. Sensitization campaigns and the provision of communication materials to household members concerned about an RRT responder returning home can be considered.^21,22^ Such measures can be implemented in the pre-deployment, deployment, and post-deployment phases to ensure ongoing communication and support is provided to the RRT responder household.

## Conclusions

Countries around the world are facing similar RRT operational challenges during their response to the COVID-19 pandemic. The COVID-19 response has demonstrated the need for fast and effective strategies, underscoring the critical need to address these RRT operational challenges to strengthen countries’ COVID-19 response efforts while ensuring the safety of their RRT responders.^5^ Although the challenges presented herein reflect solely the 12 countries requesting CDC support, they originated from diverse geographic, socioeconomic, and RRT infrastructure contexts, and thus are likely applicable to other COVID-19 response settings.

As the COVID-19 response evolves, new COVID-19 RRT operational challenges may arise; the situation will need continual assessment. For example, as the COVID-19 response continues, the ongoing re-deployment of RRT responders due to a limited RRT workforce may lead to increased responder fatigue. This may be especially true if RRT responders are performing in dual roles in the field, leading to faster burnout and negatively impacting resiliency.^34^ Additionally, there may be a greater need to repurpose RRTs and re-examine their use for critical public health programming activities that may be stalled, especially in countries facing sustained transmission and a protracted response. If countries recruit additional RRT responders, accounting for responder fatigue, their use in other public health programming, as well as any health monitoring and post-deployment isolation requirements will be essential when developing the RRT staffing plan. Additionally, countries experiencing a recrudescence of COVID-19 transmission may consider pre-positioning RRT responders sub-nationally and in certain “hotspots” (eg, urban centers, transportation hubs) to account for in-country travel restrictions and the potential for wide geographic spread.^33,50^ Monitoring changes in RRT operations and corresponding challenges as the response evolves will be paramount to ensure RRTs continue to be effective and efficient.

Regardless of future challenges, the current challenges highlighted should be addressed to ensure RRTs are contributing to effective country response strategies to mitigate COVID-19 transmission while ensuring RRT responders’ health and safety. Given the current limited literature on RRTs in the context of COVID-19, further research is needed to develop specific and tailored recommendations. Without COVID-19 RRT research currently available, the recommendations provided build upon previously published general guidance for RRT operations and management sciences, allowing implementation of these measures by countries at any stage of RRT development. With the potential applicability beyond the current COVID-19 response, the incorporation of these recommendations into a country’s RRT response plans and processes can not only improve the current RRT operations, but also strengthen the country’s overall emergency response operations preparing for future outbreaks.
